# Mixed methods evaluation of targeted selective anthelmintic treatment by resource-poor smallholder goat farmers in Botswana

**DOI:** 10.1016/j.vetpar.2015.10.006

**Published:** 2015-11-30

**Authors:** Josephine G. Walker, Mphoeng Ofithile, F. Marina Tavolaro, Jan A. van Wyk, Kate Evans, Eric R. Morgan

**Affiliations:** a School of Biological Sciences, University of Bristol, Bristol Life Sciences Building, 24 Tyndall Avenue, Bristol BS8 1TQ, UK; b Cabot Institute, University of Bristol, BS8 1UJ, UK; c Elephants for Africa, Maun, Botswana; d Department of Veterinary Tropical Diseases, Faculty of Veterinary Science, University of Pretoria, Private Bag X04, Onderstepoort, 0110, South Africa; e School of Veterinary Science, University of Bristol, Langford House, Langford, North Somerset, BS40 5DU, UK

**Keywords:** FAMACHA^©^, Participatory epidemiology, Livestock management, Goats, Targeted selective treatment, Nematodes

## Abstract

Due to the threat of anthelmintic resistance, livestock farmers worldwide are encouraged to selectively apply treatments against gastrointestinal nematodes (GINs). Targeted selective treatment (TST) of individual animals would be especially useful for smallholder farmers in low-income economies, where cost-effective and sustainable intervention strategies will improve livestock productivity and food security. Supporting research has focused mainly on refining technical indicators for treatment, and much less on factors influencing uptake and effectiveness. We used a mixed method approach, whereby qualitative and quantitative approaches are combined, to develop, implement and validate a TST system for GINs in small ruminants, most commonly goats, among smallholder farmers in the Makgadikgadi Pans region of Botswana, and to seek better understanding of system performance within a cultural context. After the first six months of the study, 42 out of 47 enrolled farmers were followed up; 52% had monitored their animals using the taught inspection criteria and 26% applied TST during this phase. Uptake level showed little correlation with farmer characteristics, such as literacy and size of farm. Herd health significantly improved in those herds where anthelmintic treatment was applied: anaemia, as assessed using the five-point FAMACHA^©^ scale, was 0.44–0.69 points better (95% confidence interval) and body condition score was 0.18–0.36 points better (95% C.I., five-point scale) in treated compared with untreated herds. Only targeting individuals in greatest need led to similar health improvements compared to treating the entire herd, leading to dose savings ranging from 36% to 97%. This study demonstrates that TST against nematodes can be implemented effectively by resource-poor farmers using a community-led approach. The use of mixed methods provides a promising system to integrate technical and social aspects of TST programmes for maximum uptake and effect.

## Highlights

• 47 farmers in Botswana were trained to use targeted selective treatment for worms of small ruminants.
• Goats from treated herds showed greater improvements in health than those from untreated herds.
• The system was adopted successfully by farmers with a range of levels of literacy and education.
• Mixed methods were used for evaluation and interpretation of the system within a cultural context.

## Graphical abstract

**Figure:**
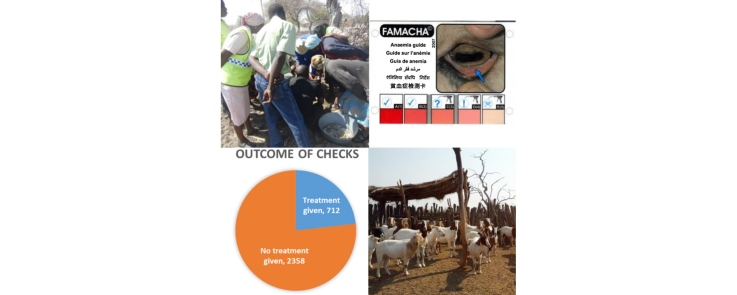

## 1 Introduction

Gastrointestinal nematodes (GINs) affect health and production in livestock worldwide by reducing the productive value of animals through declines in milk production, growth rate, fertility, and increased susceptibility to other diseases (Cobon and O’Sullivan, 1992, Perry and Randolph, 1999, Thumbi et al., 2013). In South-East Asia and sub-Saharan Africa, helminth infection is ranked as the animal health constraint with the highest impact on resource-poor livestock keepers (Perry et al., 2002).

Globally, the acute threat of anthelmintic resistance makes whole-group treatments unsustainable and has led to adoption of targeted treatment strategies in intensive livestock production systems (Van Wyk, 2001, Kenyon et al., 2009, Charlier et al., 2014). Targeted selective treatment (TST) is based on the premise that most animals are able to cope unaided even in the face of severe parasite challenge (Malan et al., 2001), so it is possible to avoid losses to the whole flock or herd by only treating the subset that are clinically affected by heavy parasite infection (Van Wyk, 2008, Molento et al., 2009, Leask et al., 2013). At the same time, parasites that are not exposed to the drug (i.e. in *refugia*) will maintain non-resistant alleles in the population, diluting the genetic contribution of any anthelmintic resistant worms which survive in the treated animals (Van Wyk, 2001). However, uptake of the selective treatment approach is limited by the reluctance of farmers to risk sacrificing short term productivity in the interests of long term sustainability (Charlier et al., 2014).

In resource-poor regions, GINs affect the livelihoods of individual subsistence farmers rather than the profit margin of large production systems. Despite small average herd sizes, subsistence farmers are unlikely to have the resources for regular whole-group treatments and face high costs of anthelmintic drugs relative to animal value. In addition, those grazing on communal pastures, as is the norm in Botswana, are not able to practise other recommended strategies to control GINs such as pasture management and rotation, and selective breeding (Krecek and Waller, 2006, Van Wyk et al., 2006, Riley and Van Wyk, 2009). TST would enable rapid gains in animal health and production for relatively small investments in chemotherapy, and an inherently sustainable approach from the outset. However, limited access to education for farmers and sparse animal health support systems could challenge the implementation of TST.

The blood-sucking nematode *Haemonchus contortus* is the number one helminth infection impacting resource-poor livestock keepers (Perry et al., 2002). TST for *H. contortus* infection can be implemented using simple indicators, and primarily the FAMACHA^©^ system, which uses ocular mucous membrane colour as an indication of anaemia caused by haemonchosis (Malan et al., 2001). This system has been implemented and validated around the world as a method for TST in both sheep and goats (Bath et al., 2001, Vatta et al., 2002, Vatta et al., 2001, Kaplan et al., 2004, Mahieu et al., 2007, Di Loria et al., 2009, Scheuerle et al., 2010, Sotomaior et al., 2012, Maia et al., 2014, Maia et al., 2015, Nabukenya et al., 2014). Both the FAMACHA^©^ system and the Five Point Check^©^ system, which includes FAMACHA^©^ and additional checks for clinical signs caused by non-haematophagic internal parasites, are designed for easy use by farmers without veterinary skills (Bath and Van Wyk, 2009, Maia et al., 2014). However, the use of these systems has been primarily studied in commercial flocks, with few studies on its application in resource-poor settings, and no investigations of the constraints or opportunities associated with the social context in which it is implemented (Nabukenya et al., 2014, Maia et al., 2015).

In this study we used a novel mixed method approach to determine the feasibility of introducing TST for sustainable and cost-effective management of GINs in small ruminants by smallholder subsistence farmers, the majority of whom had not previously used anthelmintics. Mixed methods research, where qualitative and quantitative approaches are combined to address the same aim, provides the potential to better understand how TST can be implemented within existing technical, social, and educational contexts in a way that is valuable to resource-poor farmers and sustainable (Ozawa and Pongpirul, 2014). Previous research has neglected this area, focusing instead on technical improvements in TST and validating its effectiveness for anthelmintic resistance and economics. We aimed to empower the farmers to assess and manage the health of their own livestock, thereby increasing resilience and food security. At the same time, we were able to assess the performance and benefits of TST in this setting, and understand the social context of implementation, such that we are better placed to embed TST into livestock management programmes elsewhere.

## 2 Materials and methods

### 2.1 Mixed methods framework

**Fig. 1:**
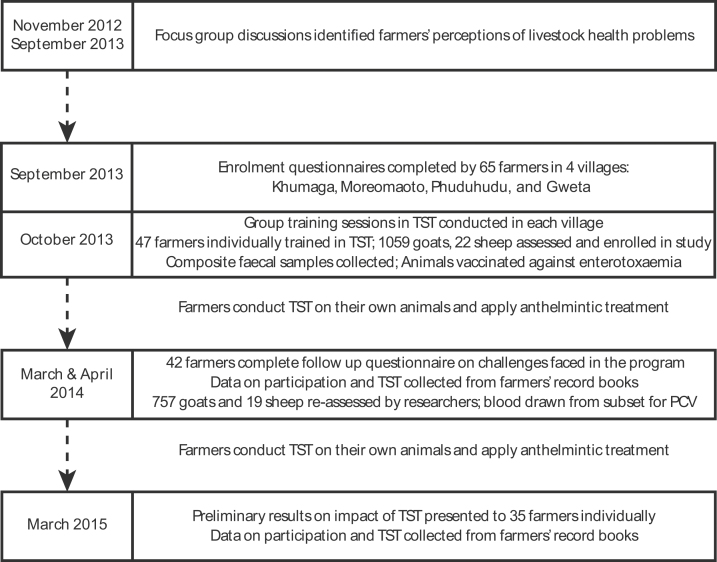
Flowchart of mixed methods, multi-phase study design. Key time points in the study are represented, additional detail on each phase is in the online Supplementary material.

We used a multiphase research design where qualitative focus group and individual interview data were collected at the beginning and end of the study, respectively, and quantitative questionnaire data and clinical data were gathered concurrently during the main part of the study. An overview of the research framework used is presented in Fig. 1, and additional information on each phase is described in Supplementary Methods in the online Supplementary materials.

Ethical approval for this study was received from the University of Bristol Faculty of Medical and Veterinary Science Research Ethics Committee (Reference 3481), the University of Bristol Home Office Liaison Team and Animal Welfare and Ethical Review Board (University Investigation Number UIN/13/043). Research approval was obtained from the Government of Botswana through the Ministry of Environment, Wildlife and Tourism and the Ministry of Agriculture, permit reference EWT 8/36/4 XXI (44).

### 2.2 Study area

The study area consisted of four villages which border Makgadikgadi and Nxai Pans National Park (MPNP) in northeast-central Botswana. The villages, Gweta (population 5304), Khumaga (758), Moreomaoto (518), and Phuduhudu (564) (Statistics Botswana, 2011), each consist of a central area and houses with yards where small numbers of domestic animals may be kept. On the outskirts of the villages, larger numbers of livestock are kept at cattle posts, which are generally made up of simple accommodation for livestock keepers, and animal folds surrounded by wooden palisade fences or thickets of thorn branches (kraals) where livestock are secured at night. Livestock, including cattle, goats, sheep, donkeys, and horses, are released in the morning to graze and return to their kraals in the evening. Total livestock ownership in these districts is approximately 65%, with 39–45% of households owning goats and 7–8% owning sheep (Statistics Botswana, 2014).

### 2.3 Enrolment, training, and data collection

The first objective of this research project was to identify parasite-related challenges to livestock production by subsistence farmers in a marginal mixed land use area shared with wildlife. We used a participatory approach to narrow the research focus to specific livestock diseases that are considered relevant to the communities. In November and December 2012 we conducted focus group discussions in Moreomaoto and Khumaga village using participatory epidemiology methods (Catley, 2006, Ameri et al., 2009, Bett et al., 2009). The two focus groups were made up of individual livestock owners who volunteered to participate after the opportunity was announced by the *Kgosi* (chief) at a village-wide meeting. The information gathered in the initial focus group discussions in both villages was then used to create an enrolment questionnaire to identify farmers to participate in the study and gather baseline information about livestock owners in the community. Participating farmers completed the questionnaire in Setswana after verbal informed consent was given; eligibility was limited to livestock owners.

During enrolment of each herd, the trainers worked with the farmers to record baseline characteristics of each animal, including species (sheep or goat), ear tag number, colour, age, sex, reproductive status, and measurement of heart girth as a proxy for weight (De Villiers et al., 2009). A composite faecal sample was collected from each enrolled herd and strongyle eggs were counted using a modified McMaster technique (Morgan et al., 2005). The farmers and trainers then examined each animal together to determine anaemia (FAMACHA^©^ score), body condition score (BCS), presence or absence of submandibular oedema (bottle jaw), and severity of diarrhoea (dag score) (adapted from Five-Point Check (Bath and Van Wyk, 2009)). The scoring method and approximate treatment thresholds used in this study are presented in Table 1.

Each farmer was asked to monitor their goats and sheep for signs of worms by applying the modified Five Point Check^©^ method approximately every two weeks, and to record the animals in need of treatment. We provided anthelmintics for the farmers to use to treat the animals designated by the system. The anthelmintic used throughout the study was 1.9% albendazole (Valbazen, Zoetis), a broad-spectrum benzimidazole anthelmintic with a short residual period. However, an albendazole–closantel combination (ProDose Orange, Virbac) was initially used in Moreomaoto. Both drugs are sold for use in sheep and goats, and were applied at the recommended dose of 2 ml/10 kg of body weight. Valbazen was readily available for purchase at government livestock advisory centres, enabling communities to continue with the TST system after termination of the study. In Moreomaoto, Khumaga, and Phuduhudu, the local veterinary extension officer was placed in charge of dispensing the drug according to the scoring criteria. However, due to circumstances beyond our control, in Khumaga the extension officer was unavailable for the duration of the study, meaning that the farmers were unable to access the drug until the follow up visit. In Gweta, each farmer was given a bottle of Valbazen due to the long distances between cattle posts. Text message reminders were sent to farmers twice per month to remind them to monitor and record the health of their flocks; messages were automated using Magpi Messenger (Magpi, 2015).

### 2.4 Measuring impact

In March and April 2014, at the end of the rainy season, follow-up surveys were conducted with 42 out of the 47 enrolled farmers. The aim of these surveys was to collect additional data to determine factors that are correlated with uptake of the targeted treatment program, and to gather feedback from the farmers on the program. Trainers visited each farmer's herd again at this time to provide refresher training to the farmer and to re-assess each animal's health using the modified Five Point Check^©^. The farmer’s record books of any checks and treatments performed between training sessions were photographed for later analysis. Some farmers were more active participants than others, in terms of the number of times they checked their flocks and the type and frequency of treatments applied. These differences in uptake of the program were used to assess the impact of TST on the health of the enrolled goats and sheep.

In a small number of goats in Gweta, a veterinary extension officer collected blood by jugular venepuncture, which was analysed for packed cell volume (PCV) to validate FAMACHA^©^ scores as a predictor of anaemia. In addition, heart girth measurement (De Villiers et al., 2009) and reproductive status were reassessed in a subset of herds. To limit the variance in girth change during the study period to a correlate of growth rate, we looked specifically at the growth of goats that were enrolled at approximately 6 months of age, using the age reported by the farmer at enrolment.

In order to quantify the impact of the program on the health of the animals, we compared each individual animal's BCS, FAMACHA^©^ score, and girth (representing weight gain) from enrolment at the end of the dry season to the re-check at the end of the rainy season. These changes were assessed in relation to the treatment type that the herd received (regardless of whether an individual goat was treated it was marked as “Selective” treatment if the herd was treated selectively), how many times that herd was treated, and a binary variable of whether the herd was treated at any point during the study period using linear regression/ANOVA. To test for robustness of the effect of treatment to confounders, we incorporated covariates in a mixed effects model with age, sex, species, reproductive status, and the non-response variable health indicator (initial BCS or FAMACHA^©^ score) as fixed effects and village as a random effect. Covariate models with and without the treatment term were compared by using a likelihood ratio test.

An improvement in FAMACHA^©^ score following treatment with anthelmintics was taken to indicate that parasitic helminths were likely to have contributed to the original presentation of anaemia in an individual. Therefore, we examined the cases of individual goats that were treated due to a high (pale) FAMACHA^©^ score and whose FAMACHA^©^ score was checked again within 30 days. All statistical analyses were conducted in R (R Core Team, 2014).

### 2.5 Uptake and feedback

Farmer characteristics were compared with measures of participation to identify patterns in uptake. Farmer characteristics included age, gender, literacy, education level, village, other employment, participation in government-run poverty eradication scheme, number of livestock carers, attendance at initial training, receipt of SMS reminders, size of herd, and previous anthelmintic use. These twelve characteristics were compared against three measures of farmer participation: whether they checked the goats themselves, number of times they checked, and whether they applied no treatment, blanket treatment only, or selective treatment at least once during the study. Pairwise tests were conducted using an ANOVA or linear regression, Fisher’s exact test, or Poisson regression. Due to small sample size, the aim was to identify factors for further investigation in a more focused way in this or future studies. Therefore, models combining multiple factors were not used, and no adjustment for multiple testing was made, to allow for the most lenient identification of farmer characteristics that might impact uptake.

Farmer feedback on the program was assessed by asking four main open ended questions: (1) What is your opinion of this program (i.e., the trainings last year and ongoing checking of livestock for signs of worms)? (2) What has been difficult for you about the program? (3) Have you seen an improvement in the health or production of your livestock since participating in the program? (4) How can we improve the program or make it easier for you to participate?

From these four questions, we received a variety of responses which we coded and combined by category. Responses to Question 1 were overlapping with responses to the other questions so we combined the results into three categories: “Challenges Faced”, “Suggested Improvements”, and “Perceived Benefits”. Farmers were also asked if they would like to continue participating in the program.

## 3 Results

### 3.1 Focus groups and enrolment

In Moreomaoto, the focus group consisted of 3 men and 9 women, while in Khumaga 4 men and 10 women participated. The full results of these focus groups are presented in Supplementary Data 1 in the online Supplementary material. For goats, sheep, donkeys and horses, participants listed worms as the most important disease problem, and also mentioned that parasitic worms are a problem for cattle. They described worm infection as seasonal, with highest levels of infection in the rainy season. Anthelmintic treatment was expensive and not available locally, so most people stated that they only treated their animals if the government provided drugs. Some people used traditional herbal medicine made from local plants to treat animals for worm infections.

Evidence of widespread worm infection was observed in faecal egg counts conducted on composite samples from 32 of the enrolled herds prior to the rainy season. The median faecal egg density (eggs per gram, EPG) measured was 700 (range 50–2400 EPG), with the 20 herds that had never previously been treated showing significantly higher egg counts than the 12 herds which farmers reported had been treated with anthelmintics within one year of enrolment (Wilcox test, *Z* = 2.28, *P* = 0.023, median untreated = 900 EPG; median treated = 325 EPG).

The individual enrolment questionnaire provided additional information on the livelihoods of the farmers in the four villages (Table 2). Most farmers (55, 85%) interviewed owned goats, while only 2 farmers (3%) owned sheep. The primary reason for keeping goats was for meat for home consumption (47, 85%), followed by to sell live for cash (41, 75%), for milk for consumption (35, 63%), to sell meat (23, 42%), for milk products for consumption (16, 29%), and use of skin and other products (8, 15%). Other reasons for keeping goats provided by the farmers were for livelihood or to help the family (12, 22%), companionship (3, 5%), and status (1, 2%).

### 3.2 Impact of targeted selective treatment

Of 47 enrolled farmers with 1059 goats and 22 sheep, 42 farmers were available for follow up on the return visit, and 757 goats and 19 sheep were re-assessed. According to farmer feedback, high mortality due to predation occurred during the study period, and 24% of farmers listed predators as one of the challenges they faced during the study.

Uptake of the system varied greatly between farmers. Twenty farmers had not monitored the health of their animals using the TST method at all between our visits, 12 checked once, and 10 checked between 2 and 9 times. Nine of the farmers who did not use TST had treated their flock. During the study period, 19 farmers only used blanket treatment (treating the whole flock), 11 farmers used a selective method at least once, and 10 gave no treatment to their flock. The two remaining farmers had unclear records and were excluded from this analysis. Factors contributing to uptake patterns, and challenges the farmers faced, are addressed in section 3.3.

TST was applied on 50 occasions (including during training sessions), which resulted in a mean treatment of 24% of each herd (S.D. 14.6%, min 1/37, max 31/49). Treatment with anthelmintics was associated with an improvement in average herd health as measured by BCS and FAMACHA^©^ score compared to untreated flocks, following the rainy season. As less than one percent of checks found submandibular oedema or a dag score above the treatment threshold, no further analysis was conducted on these indicators. No significant factors were found to explain the variance in girth change in goats enrolled in the study at 6 months of age, perhaps due to the small sample size (*n* = 49), and especially the very few goats from herds that were not treated (*n* = 7).

BCS increased significantly during the rainy season (Table 3). The mean change in BCS was 0.33, (*T* = 14.79, DF = 687, *P* < 0.0001). An increase (improvement) in BCS would be expected due to the increase in food availability during the rainy season. However, the mean change in BCS was significantly higher in herds that received treatment compared to non-treated herds (*T* = −5.8, DF = 404.5, *P* < 0.0001, mean non-treated = 0.14, mean treated 0.42, 95% C.I. of difference = 0.18 to 0.36).

**Fig. 2:**
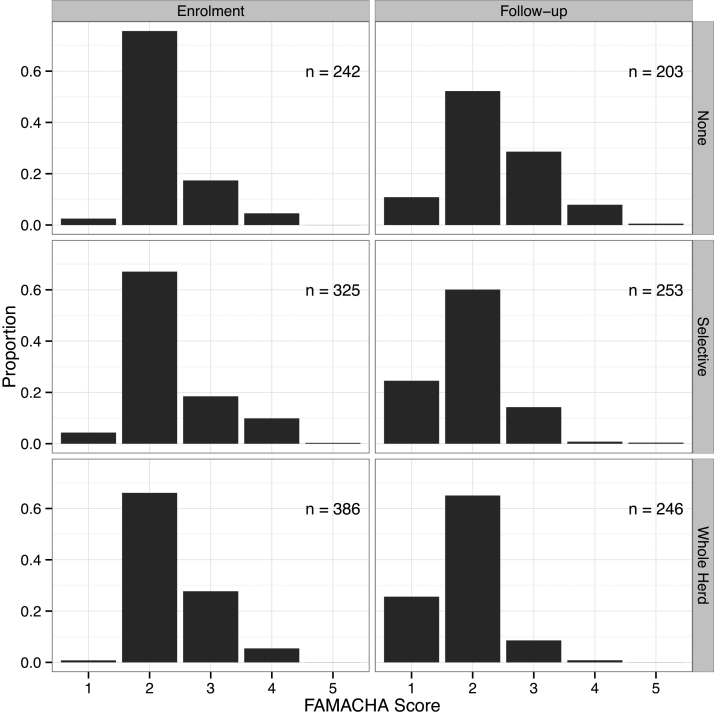
Distribution of individual FAMACHA^©^ scores at enrolment and follow up by treatment type for each herd. Low FAMACHA^©^ scores indicate higher packed cell volume (better health).

FAMACHA^©^ score decreased (improved) significantly over the rainy season (Table 3). The change in distribution of FAMACHA^©^ scores between the enrolment and follow-up visits is shown in Fig. 2. The mean change in FAMACHA^©^ score was −0.30 (*T* = −9.8, DF = 691, *P* < 0.0001). The FAMACHA^©^ score improved significantly only in goats from treated herds (*T* = 8.7, DF = 366.2, *P* < 0.0001, mean non-treated = 0.10, mean treated = −0.47, 95% C.I. of difference = 0.44 to 0.69). There was no significant difference between the different treatments (selective, whole herd) in effect on FAMACHA^©^ score.

To validate FAMACHA^©^ score as method of assessing anaemia, packed cell volume (PCV) was compared to FAMACHA^©^ score in a small sample of goats. As expected, the PCV of goats with FAMACHA^©^ score greater than 2 (“pale”, *n* = 9, mean PCV = 29.1) was lower than the PCV of goats with FAMACHA^©^ score less than or equal to 2 (“not pale”, *n* = 17, mean PCV = 32.5, *T* = 2.16, DF = 12.4, *P* = 0.051).

**Fig. 3:**
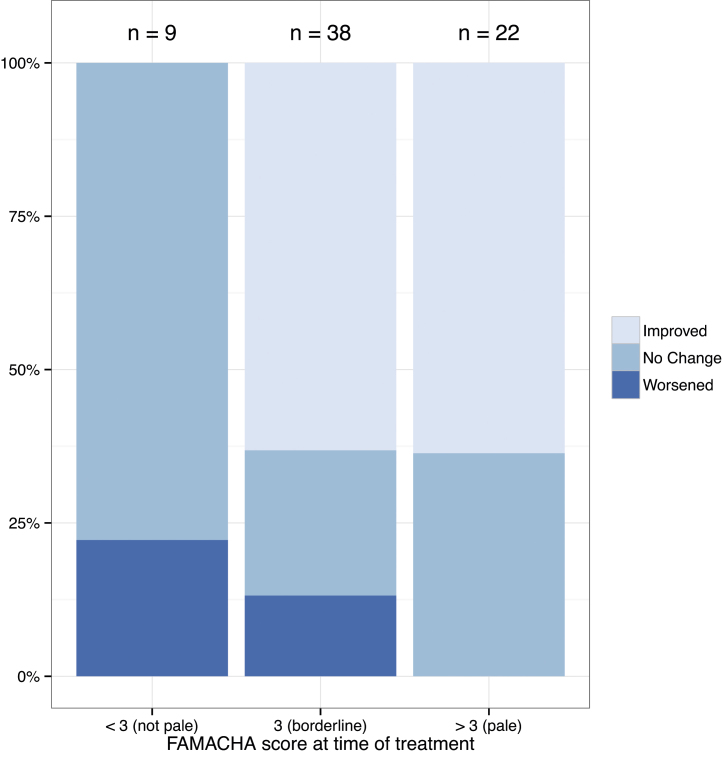
Change in FAMACHA^©^ score of individual goats <30 days after first anthelmintic treatment, by FAMACHA^©^ score at time of treatment.

To further assess the effectiveness of anthelmintic use on improvement in FAMACHA^©^ score, we assessed changes in individual goats within 30 days of treatment. We considered all the herds where FAMACHA^©^ score was checked again within 30 days of first treatment. This was the case for only 69 goats in 5 herds. The change in FAMACHA^©^ score was significantly different between the animals checked 14 days later versus those checked 24 or 29 days later, with those checked 24 or 29 days later having a greater improvement in FAMACHA^©^ score compared those checked 14 days later (lm(FAMACHA change ∼ days),), adj. *R*^2^ = 0.11, *F* = 5.20 on 2 and 66 DF, *P* = 0.008. Change in FAMACHA^©^ score after treatment was not correlated with date of treatment, kraal, sex, or age of the goat. Change in FAMACHA^©^ score after treatment was significantly correlated with starting FAMACHA^©^ score (lm(FAMACHA change ∼ first FAMACHA),), adj. *R*^2^ = 0.121, *F* = 10.35 on 1 and 67 DF, *P* = 0.002, *y* = −0.5*x* + 0.99, such that those goats with FAMACHA^©^ score of 2 that were treated were less likely to show an improvement in FAMACHA^©^ score compared to goats treated with FAMACHA score of 3 or higher (Fig. 3).

### 3.3 Farmer feedback and challenges to uptake

**Fig. 4:**
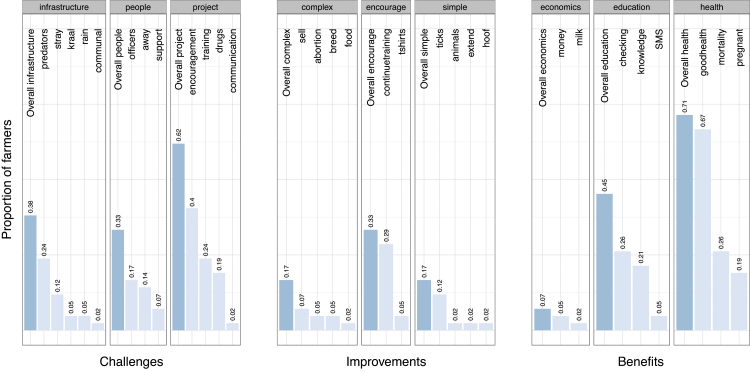
Farmer responses to feedback questionnaire, divided into three categories: *Challenges* faced, suggested *Improvements*, and *Benefits* gained. Responses in each category are further divided into three main groups of responses (see Table S1 for details of responses). Dark bars = proportion of farmers who gave answers in each response group overall; light bars = proportion of farmers who gave each individual response.

All of the interviewed farmers elected to remain involved in the program at the follow up visits in March–April 2014. All farmer responses to open ended questions regarding their opinions of the program were categorized as described in Table S1 in Supplementary Data 2 in the online Supplementary material. The proportions of farmers that gave each response are plotted in Fig. 4. Farmers were more likely to report a benefit of improved herd health if their animals were treated during the study (Fisher’s exact test, odds ratio = 21.4; 95% CI 2.5–313.8, *P* = 0.001).

Farmer characteristics for 30 respondents self-identified as primary caretakers were included in the analysis of uptake. Most characteristics were not correlated with participation. Increasing birth year (lower age) was somewhat associated with checking of goats, but the trend was not significant (lm(Checked ∼ Birth Year), adj. *R*^2^ = 0.077, *F* = 2.99 on 1 and 23 DF, *P* = 0.097). Education was not correlated with checking as a binary variable, or type of treatment strategy applied, but was positively correlated with the number of times the farmers checked their herds, with those with post-secondary education checking significantly more times than those with no formal education, primary, or secondary education only (lm(Times They Checked ∼ Education), adj. *R*^2^ = 0.38, *F* = 6.42 on 3 and 23 DF, *P* = 0.0025).

The category of treatment applied differed significantly between villages (Fisher's exact test, *P* = 0.00087), and was associated with herd size. The smallest herds were most likely to have received only non-selective treatments, intermediate sized herds to have received no treatment, and larger herds to have received selective treatments.

Of 35 farmers individually interviewed in March 2015, 8 had used selective treatment since the previous follow up in March–April 2014. This included 2 farmers who had not checked their animals using the TST system at all during the previous phase of the study. Feedback was primarily positive. Individual responses from farmers are presented in Supplementary Data 3 in the online Supplementary material.

## 4 Discussion

The mixed methods approach demonstrated in this study complements traditional parasitological investigations and allowed us to evaluate TST in terms of the social and contextual reasons for participation as well as its technical performance. The results of both the qualitative and quantitative portions of this study demonstrate that it is valuable and feasible to implement community-led TST programs for nematodes in small ruminants in a resource-poor farming context, while acknowledging the specific challenges faced by both farmers and researchers.

TST programs are valuable to resource-poor farmers in terms of improved animal health outcomes as well as knowledge of available management strategies. Most farmers (71%) mentioned that their goats were in good health following participation in the program, and were more likely to describe this as a benefit if their goats were treated. However, because the program was implemented before the rainy season, and the follow-up questionnaire was conducted after the rainy season, the good health and growth of the animals is certainly confounded by seasonal access to food following a previous drought year. BCS in particular has been found to vary seasonally in goats in Botswana (Nsoso et al., 2003). Nevertheless, BCS and FAMACHA^©^ score showed greater improvement in animals from treated herds, with equal improvements in FAMACHA^©^ in herds treated selectively compared to whole herd treatment. BCS improvement was greatest when the whole herd was treated, which may reflect that the treatment strategy was primarily driven by FAMACHA^©^. Overall, treatment with anthelmintics contributed to an improvement in anaemia and in BCS. Although the farmers in this study did not undertake regular performance monitoring, other production benefits from worm control are likely to include better growth, higher kid survival rate, and higher milk production, with significant economic gain (Rinaldi and Cringoli, 2012). Assessing these measures would provide additional insight into the improvement in production that may be achieved by using a TST system.

This study provides initial support for the validity of the FAMACHA^©^ system for assessing anaemia in the type of goats that are raised in the study area. FAMACHA^©^ score was correlated with PCV in the small sample that we assessed. Further validation using larger sample sizes would be valuable. Comparing FAMACHA^©^ scores with individual egg counts would demonstrate more precisely the link between FAMACHA^©^ and worm burden and especially the role of *H. contortus* as a cause of anaemia in the study area. Future research on the prevalence of anthelmintic resistance in the area, and whether farmers' use of TST successfully extends the usefulness of particular anthelmintics, would be beneficial to further validate this program. In addition, a more extensive study of seasonal and spatial patterns of worm burden in the area would contribute to a better understanding of the factors that influence parasite transmission and production efficiency, and focus monitoring and treatment effort on periods of high risk.

The improvements in animal health from TST will be especially beneficial to resource-poor farmers. Farmer responses in the elicitation exercises, as well as improvements in BCS and FAMACHA^©^ score following treatment confirm that GINs have a significant impact on goat health and production in the study area. This is not surprising given their ubiquitous presence and impact on grazing ruminants worldwide, including in low-income economies (Perry et al., 2002). The communities in this study rely heavily on livestock for food and income, and while there is extensive local knowledge of the broad range of diseases that might impact their livestock, including worms, the focus groups indicated a high level of reliance on government assistance in the form of vaccination and treatment for certain diseases, such as contagious abortion, anthrax, and foot and mouth disease in cattle. However, there is little government-led veterinary support for small ruminants, which are significant in local livelihoods but of little commercial importance to the national meat export industry (Botswana Meat Commission, 2008, Statistics Botswana, 2013).

Farmers in the study villages face a number of challenges to implementing animal health interventions. They have to travel between 70 and 120 km to the nearest government livestock advisory centre where anthelmintics are available for purchase. The lack of access likely contributed to the low proportion of farmers that had treated their livestock with anthelmintics prior to the study, and along with cost will affect the continued use of TST. These factors were not assessed in this study as anthelmintics were provided for free. However, the comparison of farmer characteristics with the level of uptake in the study indicated that it is feasible for farmers of any educational background, gender, age, or literacy level to participate in this type of system, and to benefit from the training. The small sample size and large number of potential confounding variables might have limited the power of the study to detect correlates of uptake, and the qualitative feedback given by the farmers revealed some barriers which were not captured in the questionnaire data. These included losing the pens needed to record the results, and a rumour that they would be asked to return or pay for the anthelmintics at the end of the study. In addition, it became clear through the interviews that for some families the primary caretaker for the livestock was not always the same person, and when the people that were trained to use the TST system were not on duty to care for the goats, then the animals were not checked.

Farmers offered a number of suggestions for ways to improve the program, mainly more regular contact with, encouragement from, and retraining by the researchers. In addition, it seemed more successful to empower the farmers directly rather than teaching them to rely on the extension officer to provide the drugs or assist them with checking the goats; when the extension officers went on leave or had other commitments, the farmers that were reliant on them did not have access to the drugs. During the individual re-trainings, we aimed to address this by providing farmers or neighbouring pairs of farmers with their own anthelmintic supply and emphasizing that they had the knowledge to try to assess the health of their goats themselves before asking for assistance. It would be beneficial if farmers were able to share their knowledge and experience with neighbours and family members that were not able to participate in the initial program, hence building knowledge, capacity, confidence and self-reliance. If possible, future similar programs may benefit from having a trainer on the ground for a longer period in order to assist farmers to become self-sufficient, or more closely integrating locally-based animal health officers within the project. Providing additional training to the most enthusiastic farmers and nominating them to act as community liaisons could contribute to the sustainability of the program.

By engaging and empowering farmers to recognise changes and signs of disease in their livestock, there is potential to increase the resilience of farmers to changing situations, including hydrological and seasonal changes in water availability, wildlife contact, and changing temperature patterns that could drive altered patterns of disease challenge. The empowerment of pastoral livestock keepers to contribute to trans-boundary disease surveillance has been suggested in East Africa (Catley, 2006). In that study, the local knowledge that livestock keepers had was shown to be interpretable by veterinarians. If sufficient smallholder farmers recognize the benefit of checking the health of their livestock in support of the productivity of their animals, this action could scale up and contribute to a system of bottom up animal health surveillance and resilient livestock management. Due to the extensive veterinary infrastructure in Botswana, where each village has local employees linked to regional veterinary offices, information gathered by local farmers would be straightforward to convey to a centralized system. The principles of TST could also be extended to other livestock, including cattle (Charlier et al., 2014), and simple diagnostic tools have been developed for a variety of diseases of cattle beyond nematode worms (Eisler et al., 2012). A locally led, active, and participatory surveillance system could lead to improved food security for Botswana's farmers as well as the European countries which import their meat products.

In conclusion, community-led TST for GINs in small ruminants was found to be feasible and effective for resource-poor farmers. Empowerment of farmers to take control of livestock disease may be used to improve surveillance and management of livestock in communal grazing areas. The use of mixed methods further improved the researchers' understanding of barriers to use of the system by the farmers, and farmers were appreciative of the collaborative way in which the program was implemented. Importantly, in the present study anaemia was reduced just as effectively in herds using TST, where on average one quarter of the herd was treated, as in those applying blanket treatments. This suggests that the approach has great potential for improving productivity and underpinning poverty reduction for subsistence farmers. The evidence presented in this paper, and the success of community-led mixed methods, should encourage efforts to embed targeted selective anti-parasitic treatment into animal health improvement programs on resource-poor farms in other areas and situations.

## Figures and Tables

**Table 1:** Targeted selective treatment method and treatment thresholds used in this study, adapted from the Five-Point Check (Bath and Van Wyk, 2009).

Check	Description	Scale	Treatment threshold	Possible infection
Eye	Mucous membrane colour measured with FAMACHA card indicates anaemia	1–5	Treat 3–5 (pale)	*Haemonchus contortus*, other parasites or conditions
Jaw	Submandibular oedema indicates hypoproteinaemia	0 or 1	Treat if present	*Haemonchus contortus*, other parasites or conditions
Back	Body condition score (BCS)	1–5	Treat if <2 (thin) in combination with poor Eye or Tail	*Teledorsagia* spp, *Trichostrongylus* spp, *Nematodirus* spp, *Oesophagostomum* spp, other parasites or conditions
Tail	Dag diarrhoea soiling scale	1–4	Treat if diarrhoea, >2	Same parasites as for BCS

**Table 2:** Responses to selected yes or no questions from enrolment questionnaire indicating uses of livestock as sources of food and income, perceptions of worm burden, and worm treatment history.

Question	Yes (*n* = 65)	%
Livestock are used as a source of income	62	95
Livestock are your primary income source	36	55
Livestock are used as a source of food	52	80
Livestock are your primary food source	13	20
Are worms a problem for your animals?	54	83
Have your animals ever been treated for worms?	23	35

**Table 3:** Effect of treatment on change in BCS or FAMACHA^©^ score. Reference category for “Treated” is False and for “Treatment Type” is whole herd treatment. Effects are model estimate (standard error); adjusted *R*^2^.

Dependent variable	Treatment variable		Effect without covariates^*^	Effect with covariates	Likelihood ratio test of treatment term in covariate model^*^
BCS change	Treated		0.271 (0.048); 0.042 ^***^	0.273 (0.066)	*Χ*^2^ (1) = 17.4 ^***^
Treatment type	None	−0.36 (0.055); 0.057 ^***^	−0.47 (0.076)	*Χ*^2^ (2) = 48.7 ^***^
Selective	−0.15 (0.053); 0.057 ^**^	−0.31 (0.055)	
Times treated		−0.009 (0.011); 0.00	−0.053 (0.012)	*Χ*^2^ (1) = 18.0 ^***^
FAMACHA change	Treated		−0.566 (0.065); 0.10 ^***^	−0.405 (0.092)	*Χ*^2^ (1) = 18.5 ^***^
Treatment type	None	0.615 (0.075); 0.10 ^***^	0.446 (0.105)	*Χ*^2^ (2) = 17.9 ^***^
Selective	0.072 (0.072); 0.10	0.104 (0.076)	
Times treated		−0.043 (0.015); 0.011 ^**^	0.0169 (0.018)	*Χ*^2^ (1) = 0.80

^*^p < 0.05.

^**^p < 0.01.

^***^p < 0.001.
