# Supplementary material for: Mixed methods evaluation of targeted selective anthelmintic treatment by resource-poor smallholder goat farmers in Botswana
Source: Vet Parasitol. 2015 Nov 30;214(1-2):80–8. doi: 10.1016/j.vetpar.2015.10.006 (PMC4671485; doi:10.1016/j.vetpar.2015.10.006)
Supplement: Supplementary file 1 [file mmc1.pdf]

Online supplementary material

Mixed methods evaluation of targeted selective anthelmintic treatment by resource-poor smallholder goat farmers in Botswana

Authors:

Josephine G. Walker[a,b,c]*, Mphoeng Ofithile[c], F. Marina Tavolaro[d,e], Jan Van Wyk[d], Kate Evans[a,c], Eric R. Morgan[b,e]

a. School of Biological Sciences, University of Bristol, Bristol Life Sciences Building, 24, Tyndall Avenue, Bristol, BS8 1TQ, UK
b. Cabot Institute, University of Bristol, BS8 1UJ, UK
c. Elephants for Africa, Maun, Botswana
d. Department of Veterinary Tropical Diseases, Faculty of Veterinary Science, University of Pretoria, Private Bag X04, 0110 Onderstepoort, South Africa
e. School of Veterinary Science, University of Bristol, Langford House, Langford, North Somerset, BS40 5DU

*** Corresponding author:**

J.G.Walker@bristol.ac.uk

Tel. +44 1173941389

Contents

Supplementary Methods: Details on research project phases 3

Supplementary Data 1: Results of focus group discussions, 2012 5

Supplementary Data 2: Table S1 12

Supplementary Data 3: Farmer feedback in individual interviews 13

Supplementary Methods: Details on research project phases

At the start of the study in November 2012, we held focus group discussions and collected qualitative and semi-quantitative data to determine which diseases the communities perceive to be a problem for livestock. GINs were identified as a large problem, so in the second phase we trained farmers to use TST for these parasites in goats and sheep. The impact of this program was measured quantitatively through parasite and clinical data and questionnaires at enrolment in September-November 2013 and follow up after the rainy season in March-April 2014. In March 2015, the enrolled farmers were contacted again and individually interviewed, during which time results from the previous year were shared with them.

Each focus group consisted of a semi-structured interview conducted in Setswana by a native Setswana speaker. The meetings in Khumaga and Moreomaoto followed the same structure defined by a topic checklist. Participants named all domestic species kept in the village, and used proportional piling to indicate their relative abundances. They also ranked the animals in order of importance or value; participants interpreted the ranking in a way that made sense to them. In addition, the groups discussed diseases that impact each livestock species. Each group then drew a map of the village and a seasonal calendar to describe spatial and temporal patterns of agricultural practices, water use, wildlife contact, and disease.

Participants contributed and reached a consensus concerning their answers through discussion; where no consensus was reached the conflicting opinions were recorded. No individual information on participants was retained beyond numbers by gender of the group, and participants were asked to answer the questions in terms of characteristics of their community rather than individual experiences.

Further meetings were held in September 2013 to expand the study to two more villages (Gweta and Phuduhudu) and to commence animal health assessment training at all locations. Volunteers were sought for the TST trial; in addition, in Gweta, livestock owners with cattle posts close to the border with the national park were identified and recruited by the local veterinary extension office.

In September 2013, 65 farmers completed enrolment questionnaires. The questionnaire asked farmers to report the numbers of domestic animals they own and for what purpose; the sources of income and food on which their households rely; opinions of and level of contact with wildlife; basic livestock management practices used; and their perception of disease problems affecting their livestock. The written questionnaire complemented and extended the earlier oral participatory discussions.

In October 2013, group trainings were conducted in three villages (Khumaga, Moreomaoto, and Phuduhudu). These trainings were open to anyone who wished to attend. Local government veterinary extension officers attended and assisted, and these key players received additional training individually, so that farmers could feasibly request assistance through pre-existing frameworks in each community.

The purpose of the training was to show farmers how to use a small quantity of deworming drug effectively by only treating those animals that show signs of worm infection. The training consisted of two parts: first, a description of GINs that infect goats and sheep, including showing a special preparation of preserved worm specimens and describing the life cycle and transmission patterns; and second, a demonstration of how goats or sheep can be examined to find signs of infection with GINs. In the fourth village, Gweta, the training was conducted for enrolled farmers on an individual basis because the long distances between cattle posts made it impractical for farmers to attend a group meeting. After open group training sessions, each farmer was visited

individually for enrolment, where the farmer reviewed the information learned at the training, and was shown how to assess his or her own goats or sheep and record the results in a provided record book. The record book used a simple, clear format with pictograms to eliminate reliance on the literacy of the recorder.

The Botswana Department of Veterinary Services recommends vaccination of sheep and goats against enterotoxaemia (pulpy kidney disease) at least 10 days prior to and up to one year before treatment with anthelmintics due to the risk of intestinal *Clostridium* spp. growth following a sudden change in nutritional status. To comply with this recommendation, we provided an anti-clostridial toxoid-based vaccine (Pulpyvax, MSD animal health) at enrolment to all farmers who had not administered the vaccine to their herd or flock within the previous six months. The vaccine was administered to the entire herd by a local government veterinary extension officer or inoculator at the first training visit. For those farmers who had recently applied this vaccine, anthelmintics were selectively applied to those goats or sheep meeting the treatment thresholds described in Table 2.

In March and April 2014, follow up questionnaires were completed by 42 out of 47 enrolled farmers. In order to quantify challenges to uptake of the program, farmer characteristics determined from the questionnaires were compared to measures of farmer participation. Farmer characteristics included age (year of birth), gender, literacy (a binary variable in response to "are you comfortable reading and writing?"), education level (reported as none/informal, primary, secondary, or post-secondary), village, other employment (reported as whether or not the respondent has duties during the day besides taking care of livestock), whether goats were received from the government as part of a poverty eradication scheme, how many people are available to help care for the goats, whether the respondent attended the initial training, whether the respondent received SMS reminders, the size of the herd at enrolment, and whether the farmer had treated for worms prior to the study. These twelve characteristics were compared against three measures of farmer participation: number of times they checked the goats themselves, whether they checked at all (a binary measure of the previous), and whether they applied no treatment, blanket treatment only, or selective treatment at least once during the study.

In March 2015, preliminary results of the program's impact were presented to 35 farmers during individual interviews, and any additional data recorded by the farmers using the TST system were collected. Farmers were encouraged to ask questions and provide additional feedback about the program as well as advice to other farmers that may participate in the future.

Supplementary Data 1: Results of focus group discussions, 2012

Facilitators: Josephine Walker and Mphoeng Ofithile (translator)

- People at meeting
  - Moreomaoto: (29th November) 3 men, 9 women
  - Khumaga: (3rd December) 4 men, 10 women
- Animals kept (in order of value/importance)

	Moreomaoto	Khumaga
Cow (kgoma)	1	1
Goat (pudi)	2	4
Sheep (nku)	3	5
Donkey (tonki)	4	3
Horse (pitse)	5	2
Dog (ntša)	6*	7
Chicken (koko)	7	6
Cat (katse)	8	8

*there is some argument in Moreomaoto about the importance of dogs and whether they should be number 3

- Proportional piling – how many of each type of animal:

	Moreomaoto	Khumaga
Cow	25	22
Sheep	6	14
Goat	15	22
Donkey	14	10
Horse	11	12
Dog	10	11
Cat	3	1
Chicken	18	8

- Reasons for keeping animals, filled cell indicates that answer was given:

	**Moreomaoto**	**Khumaga**
**Cattle**		
Milk	■	■
Plowing/farming	■	■
Sell to Botswana Meat Commission	■	
Butcher locally	■	
Sell for money (to get out of debt)	■	■
Skin traditionally used to make things	■	
Make and sell sour milk		■
Sell meat and skin		■
**Dog**		
Very important	■	
Guard livestock	■	■

Scare predators away		
Warn of danger at night		
Warning of thieves / guard homes		
Can sell puppies		
Dog can lead back to someone who died in the bush		
**Donkey**		
Plowing		
Sell for money		
Fetching water		
Hard work carrying things		
Tastes good		
Transportation		
Milk is medicinal, good for treating asthma		
**Goat**		
Sell meat		
Milk/Use milk for tea		
Use skin as a mat		
Gov't provides people with goats to get out of poverty – can get money to use for electricity, improving standard of living		
Make and sell sour milk		
Sell for money		
Eat		
**Horse**		
Herd cattle		
Transport (ride as fast as vehicle)		
Track stolen cows even if far		
Plowing		
Can sell for money		
**Sheep**		
Sell, costs more than a goat		
Use wool to make clothes		
Meat is very tasty		
Milk, make/sell sour milk, milk for tea		
**Cat**		
Keep rats away from harvest		
Take care of snakes		
Cat knows who will die and will warn by not rubbing against them anymore		
Tastes nice (a few people eat it, not all)		
**Chicken**		
Meat		
Eggs (sell/eat)		
Make feather dusters		
Sold (for a lot, female 50 pula)		
Wakes people up in the morning		
Also used by gov't to take people out of poverty		

- Diseases animals get:

	Moreomaoto	Khumaga
**Cattle**		
Foot and mouth disease (Thako le molomo) especially if buffalo come in	■	■
Anthrax (Kwatsi)	■	■
Lumpy skin disease (Mokoko malo)	■	■
Problems with joints/lameness	■	
Blindness	■	
Liver disease especially in calves still suckling (sebete)	■	
(prompted) Ticks – suck blood, transmit diseases by making wounds which get infections though them	■	
Worms (M: prompted, say problem when cows drink dirty water)	■	■
Heartwater – spread by ticks		■
Pasteurella? (serotswana)		■
Contagious abortion (pholotso) – common in drought, government administers injections to prevent		■
**Sheep and Goats**		
Mostly worms are a problem, will have diarrhea	■	■
Blindness disease – can lose almost all of the goats to this, if you don't bring them in [to treat] all the goats will die, blindness is gradual	■	
Goat goes in circles, into thick bushes, strange behaviour (Madi)		■
Wounds all over (lekgwapihi)		■
Foot rot in rainy season (thakwana)		■
Enlarged gall bladder		■
Also get abortions in sheep and goats		■
**Donkey and Horse**		
Don't have a lot of diseases	■	
Worms (dibokwana)	■	■
Abnormal growth of the hoof makes it difficult to walk (monala)	■	
Also the same blindness as the other animals	■	
Stress – fall over and die, sudden deaths (in autumn when there is nice grass coming out)		■

- What treatments and prevention do they use?
  - Moreomaoto:
    - Call vets but sometimes resort to traditional medicines/remedies, for example a tree "mokapane" [silver leaf terminalia] is used to treat worms

        - Use fever berry to rub on wounds, get worms out that are laid by flies. Flies cause problems when lay eggs on wounds made by ticks
    - Khumaga:
        - Animals are sick often, even now – have anthrax and lumpy skin. Some people buy drugs themselves but the government does some vaccination. Some do prevention, but only if they have the money. Others just wait for the government.
        - In rainy season if goats get foot rot leave them out of kraal at night
        - Other things: Dogs and cats: only rabies is a problem; Chickens get "mokorobalo", sleepy, the dies, outbreak will kill all in village, also get greenish diarrhea, wounds on eyes/blindness, and mites (matseke)
        - When prompted, say all animals get ticks. Flies transmit diseases, like anthrax and wounds in goats.
        - To kill ticks will use a powder mixed with water, and a hand sprayer, especially for cows. But because chemicals are expensive, traditional way is to make a fire and hand pick and burn the ticks.
- Information from participatory mapping exercise:
    - Moreomaoto:
        - Where keep animals at night? Each family has own kraal (these kraals are often far from the house)
        - The fence is on the far side of the river
        - During the day animals can walk anywhere in the village, all of them are branded, they cross the river and the fence
        - Wild animals come into village: lions, elephants, buffalo, hyena, zebras, wildebeest, hippos, leopard, caracal, jackal, wild dogs – but elephants and lions make the most problems, zebra and wildebeest don't cause problems, buffalo are dangerous, attack and kill people, hippos are also very dangerous and destroy crops
        - What do you think of the fence? Does it help?
            - It's not helping, it used to function but now is mostly down – predators kill livestock, elephants raid crops and take the fence down
        - How do you feel about living close to the park?
            - It's very nice to live by the park
            - It would be good if there were a barrier to stop the animals mixing
            - Get to know the lives of wild animals, learn to coexist with them
        - Where do you get water?
            - Some have pipes in yards, and there are public pipes, only fetch water from the river if other water runs out.
            - Animals only get water from the river
            - In the past the river was dry but they had wells and would dig holes by the river to make water holes from the underground water
        - Any problems of disease from the wildlife? (besides FMD already discussed)
            - Tsetse fly/sleeping sickness, but not common, mostly eradicated here
    - Khumaga:
        - Cows graze by road, walk to river to drink (unless there are ponds in the rainy season)

        - People get water from standpipes, some have own, there are some public ones
        - Wildlife staff quarters by river
        - Problems with wild animals from across the river?
            - Some come across especially elephants, lions, buffalo. Will come into village and go all the way through and cross the road onto the other side. Elephants raid crops, lions take livestock.
        - Is the fence working?
            - Now fence is damaged but it used to be effective.
        - Cows sometimes go into park.
        - Livestock are free to roam. Kraal at night but not during drought so they have more time to graze because it's too hot during the day.
        - Do you like living close to the park or is it a problem? **[this seems to be an issue of conflict with the government – they are afraid they will be moved off their land]
            - Like to be close to the park because originated around the river, river is lifeline, can catch fish.
            - Like animals and park as well as river, benefit from money from tourists coming into the park. People from village don't go into the park because you need a permit.
        - Do wildlife spread disease?
            - Buffalo spread FMD but no other problems
- Information from seasonal calendar exercise
    - Moreomaoto:
        - Worms worst in rainy season
        - Flies can cause blindness in cattle also in the rainy season
        - Goats still have some worms in dry season
        - Crops: maize, watermelon, pumpkin, (lerotse la legapul?), (ntshe), (lebelebele)
        - Wildlife come to the village when the crops are about to be harvested/when the watermelons are big
        - Some elephants come into the village in the dry season and knock down trees but lions and leopards are more of a problem then
        - Buffalo, there aren't a lot of them here, just occasional
    - Khumaga:
        - Stress and FMD are worse in Jan/Feb
        - Anthrax is worse in Sept/Oct
        - Villagers ask: are you looking at other villages too? Will you bring dewormers back next time? Or get access to a store in town to sell dewormers?

MOREOMAOTO PARTICIPATORY MAPPING EXERCISE:

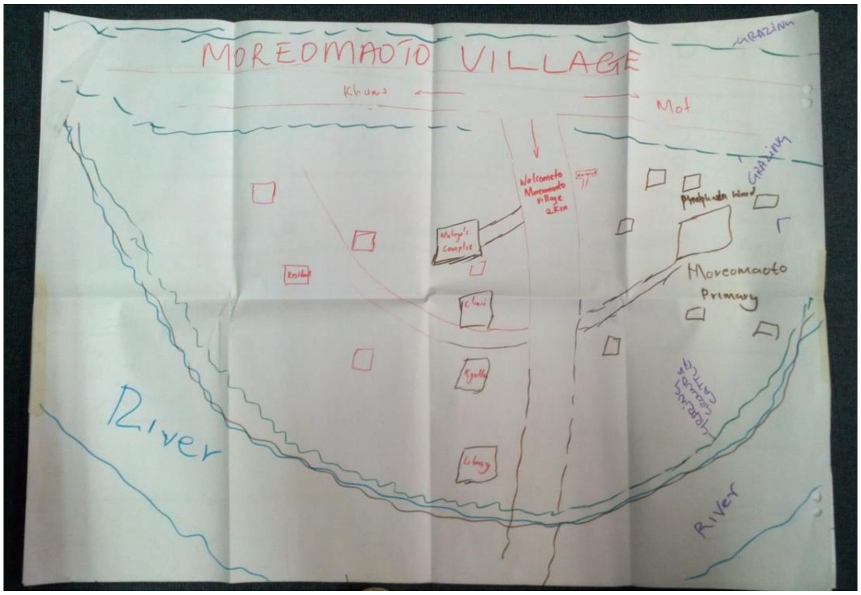

MOREOMAOTO SEASONAL CALENDAR EXERCISE

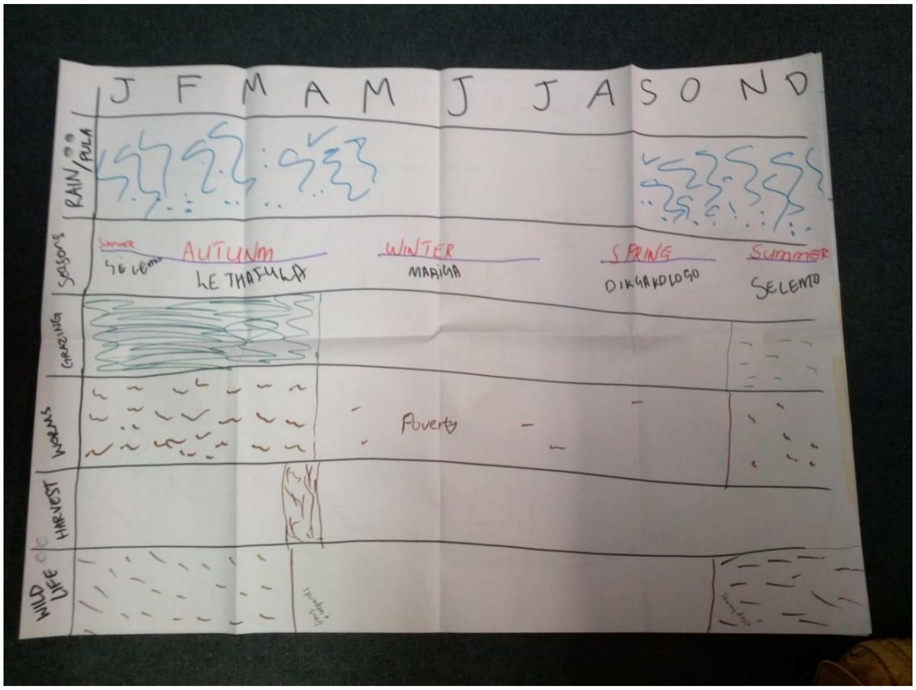

KHUMAGA PARTICIPATORY MAPPING EXERCISE

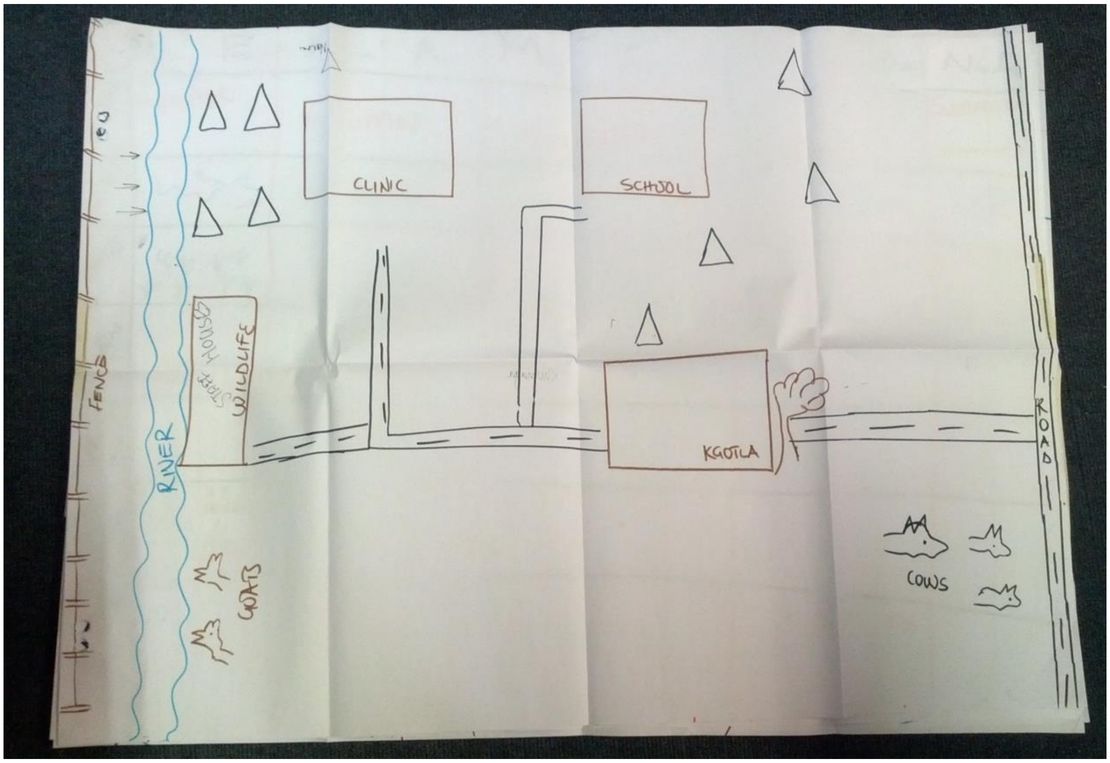

KHUMAGA SEASONAL CALENDAR EXERCISE

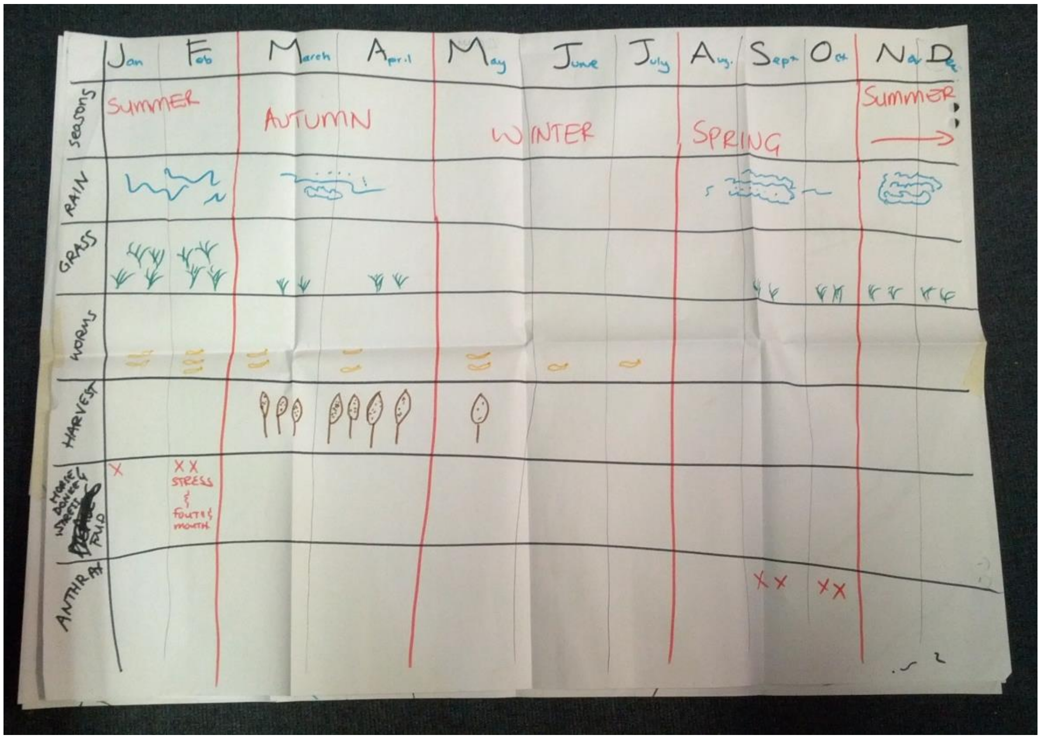

Supplementary Data 2: Table S1

Responses to open ended questions in follow-up questionnaire

Farmer feedback on the program in the follow-up questionnaire are divided into three categories: Challenges faced to implementation of the program, suggested improvements to the program, and benefits gained by participation in the program. Responses in each category are further divided into three main groups of responses. See figure 5 for proportion of interviewed farmers that gave each response.

Category	Response group	Examples of responses
Challenges to implementation	Goat-related/ infrastructure problems	**kraal:** "management of goats difficult without shelter in the kraal, some not predator proof"
		**stray:** "goats go astray sometimes"
		**rain**: "catching goats is hard in the rain"
		**predators:** "goats eaten by predators"
		**communal:** "communal grazing and mixing with other goats"
	People problems	**away:** "not able to be the person with the goats every day, difficulty with transport"
		**support**: "no one to help with catching goats"
		**officers**: "reliance on extension officers for drugs or treatment, long waits, suggest additional help"
	Project problems	**drugs**: "ran out of drugs or lack of access to drugs"
		**communication**: "couldn't reach project managers for assistance"
		**training**: "not trained, don't understand system"
		**encouragement**: "would like to be checked in on more frequently"
Suggested improvements	Complex, structural improvements requiring expert intervention	**food**: "provide food for livestock"
		**breed**: "introduce better breeds, breed improvement, artificial insemination"
		**abortion**: "medicine for contagious abortion"
		**sell**: "trouble selling goats, want market"
	Simple, attainable improvements	**hoof**: "how to trim hooves"
		**ticks**: "treat for ticks or flies"
		**extend**: "include other farmers in the program as well"
		**animals**: "deworm cows/other livestock too"
	Encouragement	**continue training**: "teach us continuously and return frequently, or have someone around all the time"
		**tshirts**: "provide tshirts"
Perceived benefits	Herd health	**pregnant**: "many goats are pregnant"
		**good health**: "goats are in good health"
		**mortality**: "reduced mortality, none died from diseases"
	Knowledge and action	**knowledge**: "benefitted from knowledge and training"
		**checking**: "checking goats is a good thing and allows us to detect other problems as well, notice sick ones earlier, build relationship with goats"
		**SMS**: "communication is good through SMS system"
	Economics and production	**money**: "saving money on medicines, not wasting medicine"
		**milk**: "increased milk production"

Supplementary Data 3: Farmer feedback in individual interviews

Note: Interviews were conducted in Setswana and translated into English, except where farmers felt comfortable speaking in English. Responses were written down at the time of the interview and may be paraphrased in parts.

Khumaga

- "This method is better than the previously used method. It's very useful to check rather than wait until the goats become very skinny and then realise they must be ill. I wish the method could be used for cows as well."
- "I was checking but I lost the pen so did not record. Now we are able to check ourselves, we learned a lot."
- "Thank you for the research. Before, the goats were always skinny and now they are healthy. You drove around to farmers and sent reminder messages which were very helpful, and if the government can take up the project in the same way it would be good for farmers."
- "The project is useful because now we can identify goats which are not healthy from those which are healthy, and know when to vaccinate/treat goats."
- Upset that when his goats were sick in 2013, he was not able to get in contact with us, and the veterinary officers were not helpful. Not happy that we were so far away.

Phuduhudu

- "Very grateful for the project because I grabbed something from it. Now I can check the goats which I didn't know how to do before. The problem is when you were away and took a long time to come back and see whether we were doing it correctly. If another person is able to come and continue the project that would be good."
- "This project is good. If it wasn't for this project by this time there would be no goats, the goats were dying before. If it could include ticks that would be good, ticks are a problem in this area"
- "I agree that there has been an improvement in health, I've noticed the treated ones are more fat than the untreated ones. It would be very sad if you don't receive any funds to continue this project. Now, as farmers in Phuduhudu we are able to check and know the health of our goats." "Thank you for coming up with this project. Because of this project there are no expenses and no goats died from diseases. Also, looking forward to seeing the project continue"
- "Very thankful for the project because it brought light to me, by now I'm starting to see the benefits. I would like to see another person come to continue from where you have stopped."
- "I want to congratulate you for coming up with the project, since you came and enrolled us in the project and treated them none have died from the disease."
- "Thank you for coming up with the project. I learned a lot from this project. I didn't lose any goats from worms but from predators. I'm looking forward to see if someone else will come to continue."
- [Veterinary extension officer] "Some of the farmers are starting to see the benefits of the project now. I learned something too and can teach the farmers."

Gweta

- "Found very much difference in health after the goats were treated"

- “No problems since the start of the project. I can still treat every year or so, have the money to do so.”
- “I appreciate the way you did the project with respect and accuracy. As we were treating the animals I learned more that I didn’t know before. Nothing more to ask but very thankful.”
- “I enjoyed working with you. What I can say is before I didn’t know how to check the health of the goats. It was my first time seeing this card, and I learned. I used to inject the animals but I didn’t know why, I just knew I was supposed to treat for worms if I had goats. Now I can check and I’m very happy about that, I can gain knowledge. In terms of continuing the project, I’m getting old and discouraged by jackals eating the goats, so I might not continue, I might give up on animal keeping. But in Botswana we rely on animals when we have no job and no pension. You really educated me with this card. Even the goats now have improved and look very healthy. The veterinary people here don’t teach us. If there is something they just explain it but it is very hard to learn. You took me and we looked at the goats together so we could try it. It’s better to do it that way to learn. We learn by practicing. I used to attend courses at veterinary services but I didn’t learn much. The best method is to go to the cattle post and try it.”

Moreomaoto

- [Veterinary extension officer] “Some farmers were just in it for the sake of being vaccinated, but others they do understand it. Some farmers were asking where you were when you were away. They have seen the difference of treating animals, and of treating only a few, because it saves medicine and money.”
- “Goats didn’t give any problem after treatment. No diarrhea, or anything, which indicates the treatment was working perfectly. I know the treatment is good, and now I’ve been given an idea of the full information I will start doing it. I appreciate the education.”
- “Goats were showing results at first, but after several weeks they had diarrhea again, so needed treatment again and many died in December, but I didn’t give Valbazen or follow up on the reminder message.”
- “I had the same problem, 4 died from diarrhea around December”
- “The medicine was used but we feared if we used the medicine too much it might kill the animals. Medicine should be tested more here. We used to treat all the animals even if in good condition, now we treat the ones in bad condition. Some rumors were going around that you would come back and want the medicine back.”
- “I would say to other farmers if projects like this come they should join so they can benefit. By the time I got in this project, they had no diarrhea or related illness. I only lost goats to crocodiles, so this project is something good for me. Some people are needing this, others wanted to join, so if the project can continue then others can benefit as well.”
